# Recalibrating neurological examination: from binary signs to probabilistic and multidimensional assessment using mobile technologies in acute neurology

**DOI:** 10.3389/fneur.2026.1901150

**Published:** 2026-09-22

**Authors:** Eugene Rezk

**Affiliations:** 1 Faculty of Informatics, Virtual and Augmented Reality Group, Institute for Visual Computing and Human-Centered Technology, TU Vienna, Vienna, Austria; 2 Entrepreneurship in Digital Health (EDITH), Medical University of Graz, Graz, Austria; 3 Trauma Center Vienna – Meidling Site, Sigmund Freud Private University, Vienna, Austria; 4 Department of Trauma Surgery, Military Hospital of the Austrian Armed Forces, Vienna, Austria; 5 Department of Medical Physics and Biophysics, Medical University of Graz, Graz, Austria

**Keywords:** acute neurology, Bayesian inference, neurological examination, multidimensional assessment, vestibular assessment, gait analysis, fall risk, mobile technologies

## Abstract

Neurological bedside signs are widely used in acute stroke evaluation as if they provide categorical evidence of focal neurological dysfunction. However, this Perspective argues that such interpretation reflects a systematic mismatch between clinical teaching and the probabilistic nature of diagnostic inference. Focusing on pronator drift and related pyramidal signs, we synthesize evidence showing that these findings generate only modest likelihood shifts and are highly sensitive to clinical context, patient state, and examiner conditions. We propose that bedside motor signs should not be interpreted as binary indicators of corticospinal tract dysfunction, but as context-dependent probabilistic signals that contribute incrementally to diagnostic reasoning. Reframing neurological examination within a Bayesian framework highlights that pre-test probability, rather than isolated signs, determines diagnostic meaning and clinical impact. This conceptual shift has direct implications for acute neurological decision-making, particularly in time-critical stroke pathways where bedside findings influence imaging and escalation decisions. We further argue that multidimensional clinical problems, including dizziness, gait instability, and fall risk, require integration of information across neurological, vestibular, musculoskeletal, visual, and behavioural domains. In this context, complementary quantitative and digital assessment approaches may provide additional functional information alongside conventional examination. By integrating probabilistic reasoning with multidimensional assessment, this Perspective challenges persistent categorical thinking in clinical neurology and proposes a framework for recalibrating how bedside signs are taught, interpreted, and supported by emerging digital technologies in acute care.

## Highlights

- Bedside neurological signs are probabilistic signals and should not be interpreted as binary indicators of focal pathology.
- Pronator drift provides only modest diagnostic value and must be interpreted within pre-test probability and clinical context.
- Musculoskeletal, attentional, and functional factors significantly influence motor examination findings, contributing to orthopaedic–neurological overlap.
- This cross-domain interference challenges the assumption that bedside motor signs reflect isolated corticospinal tract dysfunction.
- Clinical decision-making should treat bedside signs as incremental probabilistic updates rather than diagnostic thresholds for escalation.

## Perspective

Neurological examination necessarily begins with categorical descriptions: a sign is observed, absent, asymmetric, or equivocal (1). The problem arises when this descriptive classification is implicitly treated as equivalent to diagnostic interpretation. In acute stroke assessment, pronator drift, subtle upper limb asymmetry, and related pyramidal signs are frequently interpreted as indicators of corticospinal dysfunction and may influence imaging and escalation decisions (2–4). However, diagnostic accuracy studies suggest that these findings generate only modest likelihood shifts and are sensitive to clinical context, patient state, and examiner conditions (3, 5, 6). Rather than acting as lesion-specific markers, motor signs reflect distributed motor system output under physiological and behavioural constraints (7–10). The central challenge is therefore not to abandon categorical examination, but to distinguish the observation of a sign from the probabilistic inference drawn from it, with the diagnostic weight of that observation depending on pre-test probability and clinical setting (11–14)

## Why binary interpretation fails in bedside neurology

Categorical description remains useful for standardizing neurological examination, teaching clinical skills, and communicating findings. The difficulty arises when a categorical descriptor is treated as a direct diagnostic conclusion. A “positive” pronator drift, for example, may provide evidence compatible with corticospinal dysfunction, but does not by itself establish its presence; conversely, a negative finding does not necessarily exclude dysfunction when sensitivity is incomplete (3, 5, 6, 15). This distinction is particularly important in acute stroke assessment. The diagnostic value of a bedside finding depends not only on its sensitivity and specificity, but also on the pre-test probability of disease and the clinical context in which the examination is performed (11, 12). The same finding may therefore produce different changes in diagnostic probability across patients and settings. Treating such findings as binary diagnostic thresholds can obscure this variation and may contribute to premature reassurance or escalation (2, 4). The central problem is consequently not the use of categorical examination itself, but the failure to distinguish descriptive classification from probabilistic interpretation. A structured neurological examination can remain categorical at the level of observation while its diagnostic meaning is interpreted as incremental evidence within a broader clinical reasoning process (13, 14).

## Bayesian reinterpretation of pronator drift

Bayesian reasoning reframes neurological examination as probabilistic updating rather than categorical classification (13, 14, 16). Pre-test probability, derived from clinical context, symptom evolution, and risk profile, is updated by likelihood ratios associated with observed signs (11, 14). For pronator drift, clinical data indicate limited diagnostic sensitivity despite high specificity (5). As a result, even a positive finding produces limited upward revision of disease probability, while a negative finding rarely excludes corticospinal dysfunction with sufficient certainty to justify diagnostic closure. This is particularly relevant in early or fluctuating neurological presentations, where clinical signs are inherently unstable and temporally variable (3, 6). The key implication is that bedside motor signs are not decisive tests, but provide low-to-moderate diagnostic updates whose value lies in incremental calibration rather than diagnostic resolution (12, 13). Emerging bedside technologies may further complement conventional examination by providing quantitative measures of motor function that can contribute additional evidence to the diagnostic process (17).

## Motor signs as context-sensitive outputs

A central limitation of conventional neurological reasoning is the implicit assumption that bedside signs have stable diagnostic meaning across contexts. In reality, pronator drift is a composite motor behaviour requiring sustained postural control, proprioception, attentional stability, and cortical integration (6). It therefore reflects distributed motor system function rather than isolated corticospinal integrity (7–9). This has a direct epistemic consequence: the diagnostic performance of motor signs is not invariant but context-sensitive. The same observed drift may carry substantially different inferential weight depending on baseline prevalence, clinical setting, and patient condition (2–4). In probabilistic terms, this corresponds to variability in effective likelihood ratios across environments (11, 14, 16). Such variability is not a secondary source of error but an intrinsic property of motor system expression under clinical examination conditions, implying that diagnostic uncertainty is generated by the system itself rather than merely revealed by measurement (12).

## Cross-domain influences in neurological examination

Motor performance during bedside examination is frequently influenced by non-neurological factors, including musculoskeletal pain, fatigue, anxiety, joint restriction or structural impairments (e.g., shoulder pathology), and functional neurological symptoms (6, 18). These influences are not merely occasional confounders but may represent structurally embedded determinants of examination performance in routine neurological practice. This introduces systematic uncertainty into the mapping between observed motor behaviour and underlying neurological state. Conceptually, this represents cross-domain diagnostic interference, in which non-neurological systems modulate the expression of neurological signs (12, 14, 16). Failure to explicitly account for this structure risks attributing excessive diagnostic specificity to outputs generated by a distributed biological and behavioural system rather than by a single lesion-localised mechanism (7–9). The same principle becomes particularly relevant when clinical assessment extends beyond isolated motor signs to multidimensional problems such as dizziness, gait instability, and fall risk. These presentations may reflect interacting neurological, vestibular, musculoskeletal, visual, and behavioural components, making attribution to a single clinical domain inherently difficult. In such settings, probabilistic interpretation may therefore benefit not only from contextual clinical reasoning but also from complementary assessments that characterize different functional domains.

## From probabilistic examination to multidimensional decision support

The practical implication of probabilistic neurological examination is not to abandon categorical clinical teaching, but to add a second layer of interpretation. Early learners can first be trained to recognize and describe findings consistently, including whether a sign is present, absent, asymmetric, or equivocal. Subsequently, teaching can explicitly address how the diagnostic weight of the finding changes according to pre-test probability, clinical context, temporal evolution, and competing explanations. Case-based learning could reinforce this distinction by presenting similar examination findings in different clinical contexts and asking learners not only whether a sign is present, but how much it should change their diagnostic probability. For young clinicians, this second layer of reasoning could also be supported by structured decision aids that make uncertainty explicit rather than concealing it behind categorical labels (16). Such tools could integrate clinical context, examination findings, alternative explanations, and complementary measurements to provide an interpretable representation of diagnostic probability. The purpose would not be to replace clinical judgment or require formal numerical Bayesian calculations at the bedside, but to support calibration, reduce premature diagnostic closure, and make uncertainty more explicit. This approach may be particularly relevant to multidimensional clinical problems such as dizziness, gait instability, and fall-risk assessment, where neurological, vestibular, musculoskeletal, visual, and behavioural factors may interact. One potential technological direction is the integration of complementary measurements from different functional domains. For example, a recent conceptual framework proposed smartphone-based assessment combining ocular-motor measurements with inertial-sensor-based gait analysis, including measures of gait stability and inter-limb symmetry, as a potentially accessible approach to characterizing balance-related impairment (19). Such technologies could, in principle, provide complementary quantitative evidence alongside bedside examination rather than replacing it. The broader objective is therefore not to make neurological examination less structured, but to connect structured observation with appropriately calibrated interpretation. In this framework, digital tools may serve as decision-support mechanisms that help clinicians integrate heterogeneous clinical signals while preserving the central role of clinical judgment, see Figure 1.

**Figure 1:**
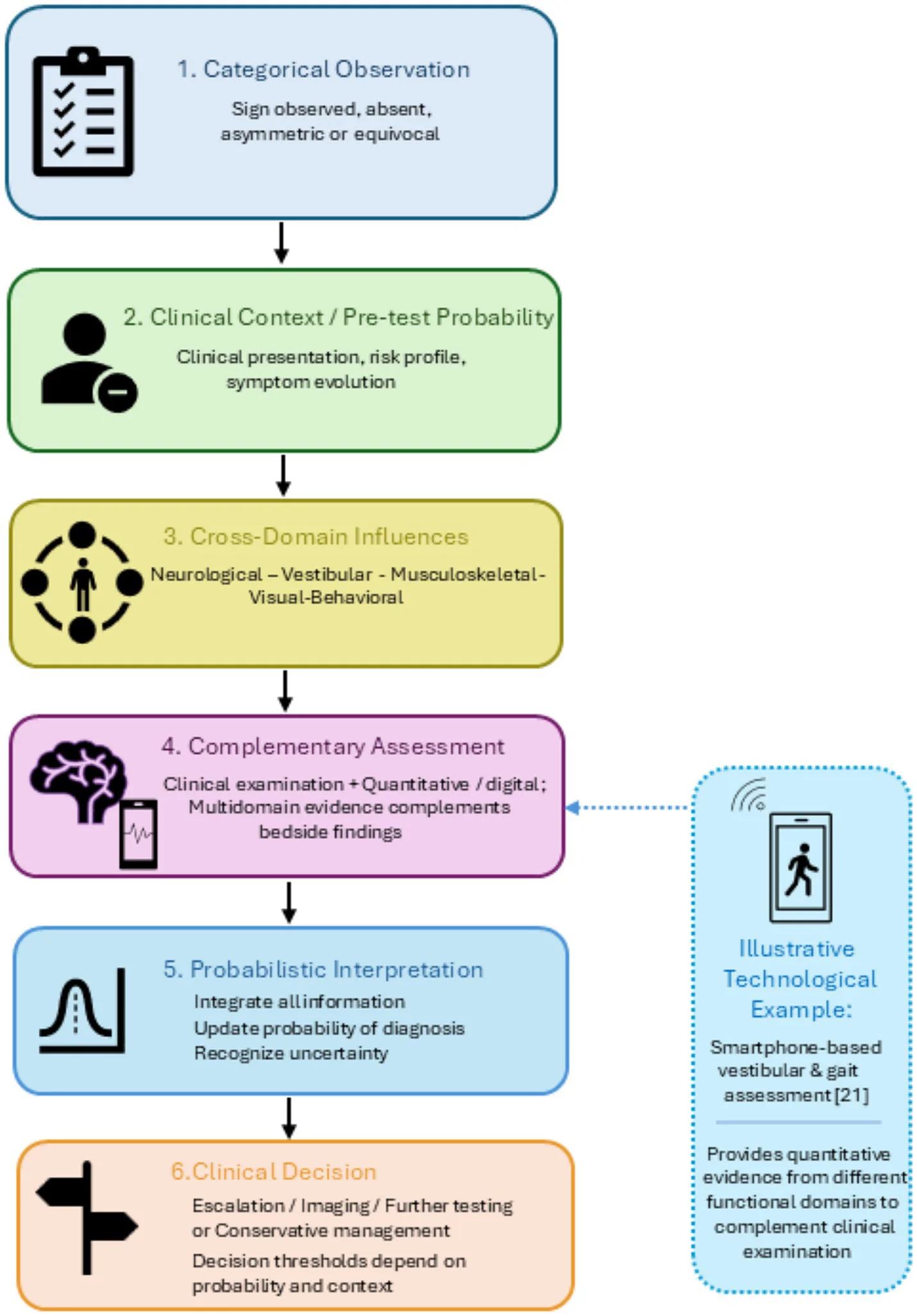
Conceptual framework for probabilistic neurological examination in acute care. Bedside findings are categorical at the level of observation but should be interpreted probabilistically within clinical context, cross-domain influences, and complementary assessments (22). Emerging digital technologies may provide additional quantitative information to support clinical decision-making (19).

## Implications for clinical decision-making

Reconceptualising bedside signs as probabilistic signals has direct implications for neurological practice and acute decision-making. First, pronator drift and related findings should not be interpreted in isolation, but integrated with pre-test probability and the broader clinical context (2, 4, 13, 16). Second, negative findings cannot reliably exclude disease when sensitivity is moderate, particularly in early or evolving presentations (3, 5). Third, the diagnostic meaning of an identical finding may vary across clinical contexts because of differences in baseline prevalence, patient condition, examiner factors, and decision thresholds (11, 12, 14, 16). Accordingly, bedside findings should be regarded as incremental evidence within a broader diagnostic process rather than as independent diagnostic thresholds for escalation or exclusion.

## Beyond pronator drift: a general principle of neurological semiology

The probabilistic and context-sensitive nature of pronator drift extends to other pyramidal and coordination signs. Reflex asymmetries, finger tapping, Babinski responses, and related tests show variable diagnostic performance and generally provide greater value when interpreted in combination rather than isolation (1, 5, 20). Composite bedside assessments such as HINTS in acute vestibular presentations likewise illustrate that even highly informative examination strategies remain probabilistic rather than deterministic (4, 21). More broadly, these observations support a shift from rigid sign-based localisation towards probabilistic inference over distributed clinical states, consistent with contemporary views of neurological dysfunction as arising from interacting systems rather than necessarily from isolated tract lesions (7–9).

## Limitations

The present Perspective is conceptual and does not introduce new patient-level data. Quantitative examples draw on published diagnostic-accuracy estimates for pronator drift and related signs, which vary across studies, populations, and settings (3, 5, 6, 18). Bayesian and decision-theoretic models simplify clinical reasoning into probabilities, whereas real-world assessment integrates temporal evolution, multimodal examination, imaging, and clinician gestalt that extend beyond formal frameworks (2, 4, 16). Similarly, the proposed use of digital and multidomain assessment should be regarded as a potential direction for decision support rather than as a clinically validated diagnostic strategy. Nevertheless, explicit modelling of uncertainty provides a useful framework for clarifying the strengths and limitations of bedside neurology in acute care (12–14).

## Data Availability

The original contributions presented in the study are included in the article/supplementary material, further inquiries can be directed to the corresponding author.
